# Family-Based Treatment for Avoidant/Restrictive Food Intake Disorder: Last-Session Reflections

**DOI:** 10.3390/bs16030325

**Published:** 2026-02-27

**Authors:** Nandini Datta, Hali Boyce, Caroline West, Anni Liu, James D. Lock

**Affiliations:** 1 Department of Psychiatry and Behavioral Sciences, Stanford University School of Medicine, Stanford, CA 94305, USA; hboyce2@stanford.edu (H.B.); anniliu@stanford.edu (A.L.); jimlock@stanford.edu (J.D.L.); 2 Department of Behavioral Medicine and Clinical Psychology, Cincinnati Children’s Hospital, Cincinnati, OH 45229, USA; cwest30@kent.edu

**Keywords:** ARFID, treatment, parent learning, self-efficacy

## Abstract

Objective: Family-Based Treatment (FBT) is an emerging intervention for youth with low-weight Avoidant/Restrictive Food Intake Disorder (ARFID), yet little is known about how parents conceptualize progress, learning, and maintenance of behavioral changes after treatment completion. Clarifying parent perspectives may inform future treatment modifications and shed light on potential mechanisms of change in FBT. Methods: This qualitative study explored parent experiences (n = 19 families) during the final session of FBT-ARFID who were treated in the context of a randomized clinical trial. Qualitative data from final-session transcripts were analyzed using reflexive thematic analysis. Results: Qualitative analyses identified four themes capturing parental reflections on learning during FBT: Progress Review, Parent Learning, Maintenance Planning, and Gratitude. Parents emphasized improvements beyond weight restoration, including increased dietary variety, reduced fear around eating, and greater flexibility at meals. Parents universally reported learning core FBT principles and increased confidence about their ability to manage ARFID in their child after treatment was completed. Conclusions: According to systematic qualitative analysis of parent reflections at the end of treatment, FBT for ARFID promotes parental self-efficacy, multidimensional progress, and meaningful parental learning related to managing ARFID symptoms in their children.

## 1. Introduction

Avoidant/Restrictive Food Intake Disorder (ARFID) is a relatively new diagnostic category, introduced in the *Diagnostic and Statistical Manual of Mental Disorders* (DSM-5) in 2013 (Bryant-Waugh & Kreipe, 2012). Although ARFID has been increasingly recognized in clinical settings, empirical research examining treatment processes and maintenance of treatment outcomes remains limited, highlighting the need for studies that investigate how treatment progress is understood and sustained. ARFID is characterized by picky eating, driven by low appetite/interest, fear of aversive consequences (e.g., getting ill or sick), and/or sensory sensitivities, typically beginning in early childhood (Thomas et al., 2017). The emotional, cognitive, and behavioral features of ARFID can contribute to severe medical and psychosocial impairment, including nutritional deficiencies, delayed development, family strain, and disruptions in social functioning. ARFID impacts an estimated 3% of youth, making it a significant public health concern (Eddy et al., 2015). Despite the growing recognition of ARFID as a serious eating disorder (ED), there are no interventions with strong empirical support identified to date. In many cases, ARFID’s enduring nature complicates treatment, requiring therapists to manage treatment outcome expectations and emphasizing the probable need for ongoing home management after treatment ends.

Family-Based Treatment (FBT), an evidence-based treatment for adolescents with anorexia nervosa (AN) and bulimia nervosa (BN), aims to support parents to change their child’s disordered eating behaviors. FBT-ARFID adapts core FBT principles to ARFID patients, empowering parents to take charge of improving their child’s nutrition, expanding variety, and introducing feared or avoided foods when applicable. The overarching goals of FBT-ARFID are to reduce ARFID symptoms to the point that they do not impair developmental domains (emotional, physical, social) and provide skills for families to continue helping their child with ARFID after treatment ends (Fitzpatrick et al., 2015). This approach encourages caregiver(s) to (a) “Externalize” ARFID (separate the child from illness related behaviors and cognitions); (b) maintain an agnostic (non-blaming) stance regarding etiology to reduce guilt or self-blame; (c) prioritize rapid behavioral change related to eating rather than putative causes; and (d) support developmentally appropriate autonomy around eating as symptoms abate. FBT-ARFID offers in-session reviews of tools, such as having a meal during session 2 for therapist feedback and observation of ARFID challenges, and the use of an “Always Sometimes Never” list for caregivers to monitor changes in food types that children are willing to eat. FBT-ARFID has shown preliminary efficacy as an intervention for youth with ARFID (Datta et al., 2023) based on data from a feasibility randomized study comparing an adapted version of FBT for children ages 5–12 years with ARFID to Usual Care (UC). This study found that FBT-ARFID was feasible, acceptable, and effective in improving weight and ARFID symptoms (Lock et al., 2019a, 2019b). For the current manuscript, parent participants were identified from a recently completed clinical trial (NCT04450771) of children (aged 6–12) with low-weight ARFID, comparing FBT with an individual treatment (Van Wye et al., 2023).

Several treatment approaches for ARFID are under development, including individual cognitive–behavioral interventions (Thomas et al., 2020) and parent-focused protocols (Breiner et al., 2024; Shimshoni et al., 2020), with preliminary support. While these treatments show promise, many rely on the patient’s self-motivation or are brief and exposure-based. These may be less suited for young children or patients with low weight, whose medical vulnerability often necessitates rapid and sustained nutritional intervention. In this context, treatments that engage and empower caregivers as agents of change may be particularly relevant. FBT-ARFID offers a developmentally appropriate framework that centers the caregiver role in nutritional restoration and the long-term management of ARFID. Importantly, FBT-ARFID conceptualizes parents not only as the drivers of initial behavioral change, but also as active participants learning skills, confidence, and illness-specific psychoeducation over the course of treatment. As such, FBT-ARFID’s success is theorized to depend in part on parents’ ability to internalize treatment principles and sustain gains once therapy ends.

Despite the central role of caregivers in FBT-ARFID, little empirical work has examined how caregivers understand treatment progress, learning, and readiness to manage ARFID independently at treatment completion. Such data may provide important insights into therapeutic mechanisms that drive change. Parental self-efficacy (PSE) in changing eating behaviors is a mechanism of change in FBT for AN (Sadeh-Sharvit et al., 2018); this is also increased in parents who received FBT-ARFID (Lock et al., 2019b). However, little is known about what parents learn and consider important during treatment (Baudinet et al., 2024; Hughes et al., 2013; Rabinowitz, 2025). To date, no research has investigated how parents receiving FBT conceptualize progress, learning, and maintenance—representing a gap in understanding about parent perceptions at the conclusion of FBT for ARFID. Thus, using a qualitative analytic approach, this manuscript describes what parents learn, reflect on, and identify as problematic areas during their family’s final session of FBT-ARFID.

## 2. Materials and Methods

### 2.1. Participants

We analyzed transcripts of the final FBT session from 19 families (out of 49 randomized to FBT) (Van Wye et al., 2023) within a larger clinical trial (NCT0377821). In this trial, participants were English-speaking children diagnosed with ARFID aged 6–12 living with their caregivers, with a baseline weight below the 10th percentile for BMI and medically stable for outpatient treatment. Participants were excluded if they had a concurrent psychotic illness or other mental illness that would prohibit the use of psychotherapy, dependence on drugs/alcohol, physical conditions known to influence weight (e.g., diabetes mellitus), prior FBT, or a recently started psychiatric medication. For the present study, the only additional inclusion criterion implemented was that the family had to have a recorded final FBT session. Further, for this project, parents were the primary “participants”. Thus, the families in this manuscript were selected because their last FBT session was recorded and available for transcription. For the remaining 30 families who received FBT, 28 completed treatment but did not have final-session recordings available, and two families did not complete a final session. Of the 19 families we include in this manuscript, n = 13 used all 14 sessions, and n = 6 completed treatment earlier than 14 sessions. Coders focused on parent or caregiver learning specifically for generating themes. In 16 families, two caregivers participated, and 3 families were single-parent homes. All single-parent homes had female caregivers. Thus, a total of n = 35 individual caregivers participated. Participant and parent characteristics are presented in Table 1. All parents and children provided consent and assent before their enrollment in the study, which received IRB approval.

### 2.2. Purpose of Data Retrieval

The final session of FBT-ARFID is designed to provide an opportunity for the therapist and family to discuss the overall treatment process and perceived progress improving restrictive eating patterns (e.g., weight gain, increased variety, increased density or amount, decreased anxiety and fear about eating). Thus, this session offers an opportunity to capture how parents consolidate treatment gains and perceive treatment effectiveness.

### 2.3. Thematic Analysis

Themes were conceptualized as recognizable patterns of shared meaning across transcripts. Themes had to be organized with a clinically meaningful concept relevant to parents’ reflections/experience of treatment. Subthemes were developed to capture related but more specific aspects of the broad theme. Theme frequency is presented descriptively and may reflect the relative salience of parents’ experiences at treatment completion. Less frequently endorsed themes were retained when they provided meaningful insight into treatment processes or anticipated challenges, and frequency alone should not be interpreted as an index of importance.

Qualitative data were analyzed by authors HB and CW using reflexive thematic analysis. Both analysts brought familiarity of FBT for ARFID, though HB had not delivered the treatment clinically. The authors first read through the raw data before developing initial codes and preliminary themes. Once open coding reached saturation (i.e., no new codes could be generated), researchers reviewed each response to ensure all codes were applied uniformly. At this point, researchers used an iterative process to collaboratively identify and resolve discrepancies in coding and establish a new unified coding system. This was used to re-code all interview responses. Three 1 h discussions were moderated by ND, and coding was discussed until complete agreement was reached and themes were judged to provide a rich and comprehensive account of parents’ perspectives of the treatment (Cohen, 1960).

Given their prior knowledge of FBT, the researchers anticipated certain concepts (e.g., parental self-efficacy) to emerge but remained open to novel or unexpected insights. No statistical software was used for qualitative coding.

Inter-rater reliability was assessed prior to consensus coding using Cohen’s kappa (κ) to quantify agreement between the two coders (CW and HB) beyond chance. Kappa values were calculated across all binary coding decisions (0 = absent, 1 = present) using the formula κ = (P_o_ − P_e_)/(1 − P_e_), where *P_o_* represents the proportion of observed agreement and *P_e_* the expected agreement by chance (Cohen, 1960). Observed agreement between the two coders was 0.85, with an expected agreement by chance of 0.49, resulting in a κ value of 0.71, indicating substantial agreement (Landis & Koch, 1977). Following this assessment, all coding discrepancies were resolved through iterative reflexive discussion, and a final consensus coding scheme was applied to the full dataset. Kappa was calculated by a third party (ND) who was not involved in coding for objectivity.

## 3. Results

Four themes emerged from the qualitative analysis of the last FBT session (Table 2); subthemes were identified within each broader theme. We review each theme in turn; quotations are presented in italics and drawn from transcripts of the final FBT session in the RCT. Modifications to quotes are in parentheses.

### 3.1. Theme 1: Perceived Clinical Progress (94.74% of Families)

This theme captured the changes or improvements observed by parents during treatment. All patients in this RCT had low-weight ARFID; therefore, weight and increased volume were often initial treatment foci. We found that ~74% of parents’ comments during the last FBT session addressed an improvement in overall growth:

“She’s definitely had a growth spurt in these 14 weeks. She’s gained the weight and the height. So it’s really been a, a good amount of progress all around.”

“There is a ways to go, obviously, but weight’s gone up.”

Variety, a second subtheme among progress, was also identified as improved by ~74% of families over the course of FBT. The following quote also identifies an ongoing need to continue the work, but the skill-building in integrating new foods has been successful.

“[Patient is] definitely eating more. She has tried so many new foods in these 14 weeks. Really proud of her for that. Definitely increased quantities of milk, regular foods. And, as you know, we’re trying to increase the fruits as well—the carrots. So yeah, all around, she’s definitely made an effort. And you know, the weight has gone up. The proof is in the pudding, so to speak. Yeah, we’re just going to continue on this path.”

Another subtheme that emerged was a reduction in fear around trying new foods, which was discussed by ~16% of families in the last session. This was described by parents as noticing less fear around tolerating new foods and sensory sensitivities. This did not address the specific fear of aversive consequences in the profile category of ARFID, as very few patients in the RCT exhibited this ARFID profile.

“[Patient] loves these same places that she was very afraid to go in, or even walk by, even walking by them because of the smell. And now she asks to go!”

Lastly, ~16% of families reviewed their “Always, Sometimes Never” list in the last session. This is a list generated collaboratively with families and the clinician in initial FBT for ARFID sessions, to provide an overview of how many foods their child is eating (Always foods), and what may be important to integrate based on what the family eats regularly and what the child may be missing in their diet (Sometimes and Never foods). Here, parents did discuss the Always and Sometimes columns growing, but also highlighted that while introducing new foods, they noticed that more foods were added to the “never” column as well:

“The [Always, Sometimes, Never List]—it’s grown a lot. In all the columns, it’s longer, but really the main two [Always and Sometimes].”

This theme broadly captures profile-specific concerns typical of ARFID—growth, limited dietary variety, sensory sensitivity, and comfort level/reduction in fear when trying new foods. While parents acknowledge “*a ways to go*” and the need to continue working on specific food groups, the emphasis on overall positive progress is evident.

### 3.2. Theme 2: Specific Parental Learning Outcomes (100% of Families)

All parents raised the topic of what they learned in FBT-ARFID during the last session of treatment. We found that ~95% of families specifically referenced psychoeducation around ARFID. This included themes of externalizing the illness from their child (a core theme in FBT transdiagnostically), the need to be consistent with presenting new foods (“*I learned I might have to present a food 20 times. It’s like, well, we didn’t get it this time, but I’m not at number 20 yet*!”), and the focus on volume of preferred foods initially to address weight *(“Oh yeah, we’ve definitely learned things—we realized that the most important thing is that she gets enough calories.”).* The following quote captures learning to externalize ARFID from the child:

“Learning about ARFID has been helpful, like, separating ARFID from [my child]. What are the things that ARFID will tell him to do, or what are the things that he might do because of the ARFID. Just being more aware of those things has been helpful, it has been helpful to just conceptualize what it is and what it means, and to separate it from him as a person, but also to not let it control things. And to know that we almost have to battle the ARFID to help [my child]”

Another part of parent learning that was brought up was a review of specific strategies parents and families learned to support their child’s eating. This included food exposures (~47%), the use of rewards (~58%), and addressing challenging mealtime behaviors (~74%). Within this, families learned which strategies worked best for them and their child, noting that not every strategy is “one size fits all” and may need to be trialed and adjusted to suit their family’s needs. For instance, one family shared: *“What I found is if you sit down with her while she’s eating, and stop all distractions, this food starts going faster.”* In contrast, other families found distractions helpful for eating*: “Allowing her some distractions while eating [is helpful]. I think the best distraction is drawing or reading. She does enjoy doing that.”* Other families may use a distraction as a reward to motivate eating: *“You get a star if you’ve eaten your dinner within 30 min. And you redeem your star on the weekend for 30 min of screen time.”* Families also acknowledged that increasing volume and variety is difficult for their child and may require patience:

“Well, I think he and I realized that like things can be really hard, and you have to sometimes push through it.”

Similarly, ~68% of parents reflected on the benefits of planning ahead to manage difficult mealtime behaviors and set expectations and consistency. Parents reflected that these practices that they instilled over the course of treatment have resulted in less arguments and negotiations in the moment:

“We set the menus for dinner and his breakfast, that way we can say ‘we’re doing this’ and there’s less arguing.”

“[Planning] is one of the big ones—I know we’re always in a rush, and she’s learned to get her snacks together the night before.”

About a quarter of parents brought up parental empowerment in the last session. This encompassed both parental understanding of their role in the treatment process (helping support increasing variety and volume) and feeling as though they were able to do so effectively. This theme is alluded to across the domains of parental learning, but was more directly addressed in 25% of families:

“You know this idea that, it’s our responsibility as parents to, you know, really make sure that [our child] tries new foods.”

“[Our role helping child] was something I’ve been worried about, but now feel like, this is manageable, this is doable, and we can work towards it.”

“It’s our job as parents to make sure she eats enough. And figure it out. It’s our job to figure out what will motivate her or entice her to get enough calories in her, because her brain doesn’t work that way, and we know that.”

### 3.3. Theme 3: Maintenance Planning (100% of Families)

We found that 100% of families discussed concerns about planning for how to manage ARFID in the future, including concerns with upcoming developmental considerations (puberty), ARFID’s chronicity, and, relatedly, apprehension around termination. Given the time-limited nature of FBT, families discussed ongoing needs within this context to continue addressing ARFID-related eating behaviors. We found that ~16% of families raised questions around puberty, such as, “*Are there certain ages where new issues or things change, like puberty, but also other ages that we should be aware of where new challenges pop up*?”, demonstrating future-oriented planning and the recognized need to continue managing ARFID-related eating behaviors. Parents reflected on their learning to identify when their child would need more support, including transitions to new environments such as school; ~21% of families discussed feeling apprehension around termination, including the absence of a therapist to facilitate discussion:

“I’d be lying if I said that I wasn’t concerned about maintaining healthy communication without someone [therapist] there to facilitate the communication.”

“I—and just feeling a little bit apprehensive. I mean, it’s definitely like mixed feelings. I don’t know why I’m like getting emotional. But I—I just feel like this process has been so helpful. Just kind of like changing the dynamics right?”

“I kind of get nervous about keeping the momentum going. Especially with school starting and things just getting crazier. And it’s just, you know, making sure to stay disciplined as parents to follow through with what we said we were going to do…Yeah, just makes me nervous without being able to check-in.”

Despite this apprehension, the majority (~90%) of families identified that having family meetings and continuing to implement strategies would be helpful, taking the structure from FBT and integrating it in their own home:

“During family meetings we can discuss the great successes and things we appreciate, like, the successes around ARFID. It’s just a matter of putting it on the calendar”

“When I told [my child] we were going to stop doing this therapy, I was just like, we’re going to continue the work. But it’s just going to be a few minutes out of our day. We don’t want it to take over our lives like this doesn’t have to be a big thing, but we do want to be able to track progress”

“We can’t probably do like an hour of the 3 of us meeting, but, like you know, on Sunday, we still will meal plan, and then I feel like we should find another time in the week to just kind of touch base on all this stuff.”

ARFID chronicity (discussed by ~78% of parents) was tied into discussions around how parents will need to continue supporting their child. Several parents reflected feeling as though the differences in their child’s eating may not be “curable” but that they would be able to help them grow and eat a sufficient variety of foods to support adequate nutrition. This acceptance reflects, in part, parental empowerment, despite acknowledgement of the ongoing need to support their child.

“I think [patient] has gotten more comfortable trying a couple of things; I am proud of her efforts. I don’t think she’s cured, but I think she’s made a lot of progress.”

“But yeah, it just seems more and more likely there’s no like cure-all. And I think that’s okay.”

### 3.4. Theme 4: Gratitude (36% of Families)

About a third of families explicitly shared gratitude for the learning and sessions, reflecting that the sessions and framing of ARFID had helped “*change their thinking*”, provided a space to “*talk through some things*,” and to validate their experience of ARFID.

“I feel like it’s been good for our family. I’m thankful to you, and I am thankful for the study. And I—I hope that we continue [to make progress]. You know, his whole life, he has been this way, ever since he was small. We didn’t know what it was or what was going on. It’s just been really nice to have an actual, tangible [name].”

“I think this was a really great experience, and this has been by far the most helpful; we’ve gotten more progress out of this than anything else we’ve tried so far. So this has been great.”

“I’m not sure where [my child] would be if we hadn’t started this program with you guys. We’re grateful for this time here, because it has healed [my child].”

Overall, families reported substantial learning at the conclusion of FBT, including changes they planned to continue at home, internalization of core FBT tenets (externalization and parent self-efficacy), understanding around the possible chronicity of ARFID, and foresight around upcoming challenges (e.g., puberty or school).

## 4. Discussion

This study explores how parents receiving Family-Based Treatment for Avoidant/Restrictive Food Intake Disorder conceptualize their progress and learning as reported during their final treatment session. Results highlight that parents can separate the illness from their child (i.e., externalize) and report feeling empowered and having skills to continue managing ARFID at home. Furthermore, parents cite improvements in restrictive eating behaviors in their child—including increased range and volume of intake and willingness to try new foods. Overall, these data highlight parental learning, observed behavior change, and anticipated challenges as parents plan to continue skill-building post-treatment.

The themes identified in qualitative analyses underscore that parental views of progress within FBT for ARFID are a multidimensional construct. Parents emphasized that improvement extends well beyond weight restoration to include increased variety, reduced fear and distress around eating, and greater emotional and behavioral flexibility during meals. Parental views of progress were also reflected in parents’ growing sense of efficacy and skills to manage ARFID-related challenges. Parent learning and maintenance planning emerged as the most universal themes, highlighting the central role of caregiver skill acquisition and confidence in this treatment model.

These findings are consistent with objective data on FBT in anorexia nervosa (AN) (Lock & Le Grange, 2015), which have found caregiver self-efficacy as an important driver of change (Sadeh-Sharvit et al., 2018). Prior work in FBT for adolescent AN has demonstrated that increases in parents’ self-reported self-efficacy around renourishing their child are associated with improved treatment outcomes (Byrne et al., 2015,), supporting the hypothesis that parent confidence and skill acquisition are critical drivers of treatment success. Our findings extend this literature to ARFID, illustrating how parents conceptualize and reflect on progress not only as weight restoration but also as their own confidence in managing ARFID over time.

One consideration is the meaningfulness of theme frequency. All FBT sessions are unprompted/unscripted dialogues between the therapist and family members. Thus, variation in theme frequency may reflect differences in what parents uniformly perceive as more important learning points across treatment, rather than differences in importance, consistent with qualitative analysis principles (Braun & Clarke, 2021; Guest et al., 2012). However, less frequently endorsed themes may still capture meaningful information about how treatment is perceived. For example, the subtheme “Apprehension” was endorsed by 4 families, or 21.05%, and “Specific Strategies” was endorsed by 17 families, or 89.5%. Although less frequently endorsed, “Apprehension” provides clinically relevant insight into parental planning for maintaining treatment gains and may warrant explicit discussion during the final session of FBT.

However, themes also included areas where parents perceived a need for additional support. For example, parents expressed uncertainty about maintaining gains without therapist facilitation, including sustaining momentum and adapting FBT strategies to developmental challenges without clinical guidance. This suggests that an important future direction is longitudinal assessment of the durability and maintenance of parental learning and confidence, coupled with sustained improvement in the child’s eating behaviors following treatment completion, to determine whether these initial gains are sustained over time.

Strengths of this study include a cohort of parents randomly assigned to FBT-ARFID, strong inter-rater reliability, and a novel focus on how families articulate progress and consolidate learning during the final session of FBT for ARFID. Several limitations should also be acknowledged. First, the modest sample size limits the generalizability of these findings. Second, because the data were drawn from a randomized clinical trial involving young children with low-weight ARFID, results may not fully translate to families of older youth or those for whom weight restoration is not a primary treatment target. Additionally, families were aware that the trial consisted of up to 14 sessions and therefore anticipated treatment termination, which may shape the content or tone of their final-session reflections. Although this structure may differ from some real-world clinical settings, our outpatient program predominantly delivers time-limited, evidence-based interventions. Further, qualitative results suggest that parents found this fixed intervention sufficient to help them make substantial progress in helping their child and manage ARFID symptoms after treatment. Lastly, there are limitations inherent to a qualitative study design, including reliance on retrospective self-reporting, potential social desirability and recall biases. Future studies would benefit from systematic and prospective assessment of parent learning during treatment using objective measures, allowing for more rigorous examination of how parental learning develops and relates to treatment outcomes.

The results of this study provide an opportunity to better understand parental perspectives on FBT-ARFID at the end of treatment. As a descriptive, exploratory, and qualitative study, it is an initial report about how families receiving FBT for ARFID understand their progress and learning at treatment completion. Importantly, given the range of behaviors associated with the ARFID profiles, families highlighted gains beyond weight restoration—including increased variety, reduced fear, and greater parental confidence in managing ARFID—reflecting multidimensional skill internalization and parent learning. Parents also noted that improvement in dietary variety sometimes coincided with the emergence of new food avoidances, underscoring the evolving nature of ARFID. Findings suggest that FBT effectively promotes early change and parental learning for children who experience low weight as part of the clinical presentation of ARFID. These parental learning themes also identify possible ways to modify or adapt FBT to increase emphasis on maintenance planning, developmental considerations, and the generalization of FBT skills to novel situations and circumstances likely to arise after treatment ends. In summary, FBT-ARFID appears to promote meaningful parent learning and behavioral change beyond weight restoration, while revealing important opportunities to provide maintenance support after treatment ends. These can include offering a parent booster session or creating a concrete plan collaboratively in the session for parents to take home.

## Figures and Tables

**Table 1:** Demographics.

Variable	Child n = 19	Caregiver 1 n = 19	Caregiver 2 n = 16
*Age M (SD)*	8.94 (1.72)	40.16 (5.38)	42.50 (6.03)
*Ethnicity N (%)*			
Hispanic	4 (21.1)	3 (15.8)	2 (12.5)
Non-Hispanic	15 (78.9)	16 (84.2)	14 (87.5)
*Race N (%)*			
White	10 (52.6)	12 (10.5)	12 (75)
Black	1 (5.3)	-	-
Asian	2 (10.5)	1 (5.3)	2 (12.5)
American Indian or Native Alaskan	2 (10.5)	2 (10.5)	1 (6.3)
More than one race	4 (21.1)	4 (21.1)	1 (6.3)
*Gender N (%)*			
Male	12 (63.2)	1 (5.3)	16 (100)
Female	7 (36.8)	18 (94.7)	0
*Family Type*			
Intact	13 (68.4)	-	-
Divorced	4 (21.1)	-	-
Other	1 (5.3)	-	-
*Comorbidities N (%)* ^1^			
Yes	9 (47.4)	-	-
Attention Deficit Hyperactivity Disorder	4 (21.1)	-	-
Generalized Anxiety Disorder	2 (10.5)	-	-
Separation Anxiety Disorder	1 (5.3)		
Specific Phobia	1 (5.3)		
Oppositional Defiant Disorder	1 (5.3)	-	-
Echolalia	2 (10.5)	-	-

^1^ N = 2 participants had more than one comorbidity; thus, the sum of the comorbidities listed is greater than 9.

**Table 2:** Themes.

Theme/Subtheme	N (%)	Quotes
***Progress Review***	**18 (94.74)**	**Overview of changes families observed**
Weight	14 (73.68)	“She’s definitely had a **growth spurt** in these weeks and gained the height and weight.”
Variety	14 (73.68)	“I mean her **willingness to try things** and the variety of things that she has eaten has been huge.”
Fear Reduction	3 (15.79)	“And now she loves these same places that she was very afraid to go in or walk by because of the smell.”
ASN List	3 (15.79)	“**The list has grown a lot**, in all of the columns, but especially the always and the sometimes.”
***Parent Learning***	**19 (100)**	**Parents learning across domains**
Psychoeducation	18 (94.74)	“I think that learning about ARFID was really helpful in like what are the things that ARFID will tell him to do and just **separating the ARFID from [my] CHILD**.”
Exposures	9 (47.37)	“Learning that **I might need to present a food 20 times** before she’ll try it. It’s like, well I didn’t get it this time, but I’m not at number 20 yet.”
Rewards	11 (57.89)	“The **point system [rewards] eating thing seems to work**. We’ve had success with that. So that’s been good”
Mealtime Strategies	14 (73.68)	“Feels more manageable when it’s **broken down** into, you know, **weekly goals** or something that we’re working for. Instead of just like you need to eat, you need to eat.”
Planning Ahead	13 (68.42)	“We set the menus for dinner and his breakfast, that way we can say “we’re doing this” and there’s **less arguing**.”
Self-efficacy	5 (26.32)	“This was something I’ve been worried about, but now feel like, **this is manageable**, this is doable, and we can work towards it.”
***Maintenance***	**19 (100)**	**Reflections/concerns on managing ARFID behaviors**
Warning Signs	3 (15.79)	“If it takes him longer than 30 min to eat, I can tell that’s what’s a big hint.”
Specific Strategies	17 (89.47)	“We’re going to continue to **work on trying new foods** outside of mealtimes, even if it’s just a couple of minutes each day, just to **normalize it** and not make it a big thing.”
Apprehension	4 (21.05)	“I’d be lying if I said that I wasn’t concerns about maintaining healthy communication without someone [therapist] there to facilitate the communication.”
Developmental Considerations	12 (63.16)	“Are there certain ages where new issues or things change like puberty but also other ages that we should be aware of where new challenges pop up?”
ARFID Chronicity	15 (78.95)	“It just seems like there’s no cure at all and that’s okay. **It’s our job to figure out what will motivate her** because her brain doesn’t work that way.”
***Gratitude***	7 (36.84)	**Appreciation for therapist/sessions** “Meetings with you has been a gift and it clearly needed to happen with you in this way. Thank you for everything.”

## Data Availability

The data presented in this article are not available because they are transcripts from therapy sessions, and remain confidential. No new data were created for this project. Data sharing is not applicable to this article.
